# Unveiling allelopathic dynamics and impacts of invasive *Erigeron bonariensis* and *Bidens pilosa* on plant communities and soil parameters

**DOI:** 10.1038/s41598-024-57552-7

**Published:** 2024-05-03

**Authors:** Mohamed A. Balah, Abeer Al-Andal, Asmaa M. Radwan, Abd ElRaheim M. Donia

**Affiliations:** 1 Plant Protection Department, Desert Research Center, Cairo, Egypt; 2 Department of Biology, College of Science, King Khalid University, Abha 61413, Saudi Arabia; 3 Botany and Microbiology Department, Faculty of Science, Girls Branch, Al-Azhar University, Cairo, Egypt; 4 Medicinal and Aromatic Plants Department, Desert Research Center, Cairo, Egypt

**Keywords:** Invasive weeds, Invasion intensity indices, Allelopathy, Leachates, Decaying residues, Phenolic acids, Volatile oils, Agroecology, Invasive species

## Abstract

Invasive alien species are becoming more and more prevalent worldwide, *Erigeron bonariensis* and *Bidens pilosa* are two invasive species of Asteraceae in Egypt. To mitigate their detrimental effects and understand their differences in invasiveness, we compared the allelopathic potentials of *E. bonariensis* and *B. pilosa* using leachates, decaying residues, and volatilization processes. Notably, the allelopathic variances in leachates were significant, influenced by plant types, concentrations, and response patterns of target plant traits, as indicated by EC_50_. The relative phytotoxicity of the invasive species decayed residues peaked between 20 and 25 days in the soil, with a positive correlation with concentrations and soil properties. The highest quantities of phenolic acids were chlorogenic acid and caffeic acid reaching (5.41 and 4.39 µg g^−1^) *E. bonariensis* and (4.53 and 4.46 µg g^−1^) *B. pilosa*, in leachates extracts respectively, while in the soil extract of decayed residues were coumaric acid and ferulic acid measuring (1.66 and 1.67 µg g^−1^) *E. bonariensis* and (1.47 and 1.57 µg g^−1^) *B. pilosa*, respectively. Using GC/MS analysis, the main volatile components in *E. bonariensis* were 1, 8 cineole (5.62%), and α-terpinene (5.43%) and iso-Caryophyllene (5.2%) which showed the greatest inhibitory effects. While B*. pilosa* main constituents were trans-sabinene (5.39%) and Camphene (5.11%), respectively. Finally, the high invasion level displayed from *E. bonariensis* (0.221) compared with *B. pilosa* (0.094) which correlated with the stronger allelopathic activities against plant species, and soil properties. Therefore, the allelopathic potentialities of these species are critically relevant to their invasion success.

## Introduction

Family Asteraceae is the largest flowering plant^1^**,** which produces the most troublesome invasive weeds worldwide^1–4^, and exerts common ecological impacts on invaded ecosystems^5^. *Erigeron bonariensis* (L.) Cronquist (Asteraceae)*,* originally described in Argentina, is believed to be native to the more temperate parts of South America^6^, it is an opportunistic invader of subhumid, and subtropical pastures^7^. *E. bonariensis* is listed as an agricultural and environmental weed^8^. It is one of the most difficult weeds to control in minimum tillage farming systems which has doubled fallow weed control costs^9^. It has been identified in orchards, vineyards and roadsides in Egypt, Japan and South Africa as a resistant weed to paraquat herbicide^10^. *Bidens* genus (Asteraceae) contains about 280 species and it is common in both field crops and wild areas due to its rapid growth, and strong invasive nature^11^. *Bidens pilosa* L. is an annual plant originating from South America and widely found in tropical and subtropical areas of the world^12^. The species possesses hardiness, explosive reproductive potential, and an ability to thrive in wide environmental conditions, so it is one of the worst invasive species in Egypt^13^. *B. pilosa* has a negative effect on the native flora and is located in five governorates (Qalyubiya, Al-Sharkya, Al-Dacahlya, Cairo and Giza) in Egypt^14^. In Africa *B. pilosa* is recorded as a weed in 20 countries; it is one of the most noxious annual weeds in East Africa^15^. It is a major crop weed, a threat to native fauna, and a physical nuisance^16^. The majority of the invasive plant species produce allelochemicals with the potential to negatively affect native plant performance^17^. Invasive grasses have a competitive advantage over other members of the same family due to allelopathy and their specific allelochemicals^18^. Therefore, measures toward preventing biological invasions and biology knowledge to facilitate successful management are needed^19,20^.

In natural environments, allelopathy has been implicated in plant invasions, which is a major ecological problem^21,22^. The release of allelopathic compounds is one of the potential drivers of plant invasion^23^. Allelopathy has long been thought important as a mechanism for plant invasiveness^24^. Invasive plants excel in their new ranges because they produce new metabolites to which native species possess little resistance^25^. Invasive plants can affect native plants through competition or allelopathy^26^. The allelopathy of *Imperata cylindrica, Solidago canadensis* and *Solidago altissima* may support its invasiveness, naturalization and formation of large monospecific stands^27,28^. The successful invasions depend on interactions between introduced plants and native plant communities^29^. Allelochemicals are released in both natural and agricultural systems by leaching, root exudation, volatilization, residue decaying, and decomposition^30,31^. Allelochemicals that are released from the decayed litter can hinder the physiological and biochemical processes of seed germination^32^. Phytotoxic chemicals influence soil properties and nutrient availability, population and community structure, and weed invasion^23^. Allelochemicals released by decaying plant residues can regulate the soil microbial community and chemical and physical properties of the soil^34^. Secondary metabolites produced by alien plants may be allelopathic; if they enter the soil, they may be transported by agricultural activities, negatively affecting crop yield and biodiversity^35^. There are two possible sources of allelochemicals of plant residues; the compounds can be released directly from plant litter or they can be produced by microorganisms that use plant residues as a substrate^36^. Plant volatile organic compounds VOCs vary by species, and they are related to the abundance of neighboring plant species and plant species composition^37,38^, Allelopathic effects of VOCs participate in plant growth, competition, resistance of diseases and insect pests^39^. Therefore, it is essential to compare the allelopathic effects of invasive species to correctly estimate the phytotoxic effect of invasive species on their invasiveness^40^.

To preserve agriculture resources from invasive species and determine the necessary measures, we should identify the biological traits of invasive species and their negative impacts on the native species. Despite the number of allelopathic studies about *E. bonariensis* and *B. pilosa*, little attention has been given to comparing the allelochemicals ways of getting into the environment which has an important ecological role in their invasion. So, these studies greatly deal with two Asteraceae invasive species leachates, decayed and volatile processes and their impacts on the relevant species, and soil properties and their relationship with their invasive nature to determine leading action toward their sustainable management. Therefore, we hypothesized that among the invasive species of the same family, *E. bonariensis* was more invasive than *B. pilosa* due to their strong allelopathic potentials that were related to phenolic compounds in both aqueous leachates and decayed residues. These allelochemicals differed in both qualitative and quantitative as well as impacts on the native plant traits, diversity, and soil properties. The study addressed the allelopathy and biological characteristics of *E. bonariensis* and *B. pilosa* against Zea *mays* crop and their relevant weeds via leachates of aqueous extracts, decayed residues and volatile compounds and determined the impact on soil properties during the decaying of invasive weed residues.

## Materials and methods

### Plant material

Invasive *Erigeron bonariensis* (L.) Cronquist and *Bidens pilosa* (L.) were harvested in the wild before the flowering stage during 2020–2022, from Al-Beheria and Al-Qalibia governorates, Egypt respectively. The identification was confirmed by plant specialist Dr. Emad Abdel-Kader Desert Research Center, Cairo, Egypt. A voucher samples of *Erigeron bonariensis* (CAIH-16-9-2020-C) and *Bidens pilosa* (CAIH-11-7-2020-B) has been deposited at the Herbarium of DRC, Cairo, Egypt. The plants were dried in the shade, chopped, ground into a fine powder, and then stored in paper bags at room temperature. *Convolvulus* arvensis L., *Portulaca oleracea* L*.*, and *Echinochloa crus*-*galli* (L.) P.Beauv. were collected from *Zea mays* field in Maryut research station, Desert Research Center. These species represent monocots and dicots to detect the response pattern to allelopathic potentials of *E. bonariensis* and *B. pilosa* species.

### Analysis of *E. bonariensis *and *B. pilosa* communities within invaded sites

Primary surveys were conducted about *Erigeron bonariensis* and *Bidens pilosa* invasive weeds at 15 and 5 governorates of Egypt in random patterns during 2020 and 2022 to identify the associated community according to Thomas et al.^41^. The plant species were counted across quadrates (100 × 100 cm^2^) of invaded sites. The data of assemblages were then presented in density (plants m^−2^) for usage as a function of richness and diversity. The invasion level was represented by the invasion intensity index (*III*) = *P*_*i*_***/****MaxP*_*i,*_ where *P*_*i*_ represents the observed relative abundance of alien species in one surveyed quadrat and *MaxP*_*i*_ represents the maximum relative abundance of alien species among all surveyed quadrats, respectively^42^. Richness, Shannon–wiener index, and Simpson index of diversity were measured according to Margalef^43^, while evenness was quantified according to Pielous^44^, and Magrurran^45^ to provide more information about individual distributions**.**

### Leachates of *E. bonariensis *and *B. pilosa* allelochemicals

The aboveground parts of *E. bonariensis* and *B. pilosa* were extracted by soaking 200 g of the ground parts in 1000 ml of distilled water. This mixture was placed on an orbital shaker at 160 RPM for 12 h at laboratory temperature. Then the extract was strained through cheesecloth to remove plant materials, centrifuged at 3000 RPM for 15 min, filtrated and sterilized using a 0.22 µm pore micro-filter before bioassays. To determine the allelopathic compounds, these water extracts were acidulated to a pH value less than 5 and then partitioned with three equal volumes of ethyl acetate. The resulting ethyl acetate extracts, after evaporation to dryness, yielded residues, which were stored in the deep freezer until bioassay and analysis.

### Decayed of *E. bonariensis *and *B. pilosa* materials in soil

The dried vegetative tissues of *E. bonariensis* and *B. pilosa* were incubated in sandy soils, chopped into pieces smaller than 1 cm, at 0%-, 1.25%-, 2.5%-, and 5%-gram dry weight per 100 g soil for durations of 0, 5, 10, 15, 20, 25, 30, 35, 40, 45, and 50 days. Plastic pots 15 cm in diameter and 14 cm in height were filled with sandy soils (1 kg), and their moisture was adjusted to 70% of water-holding capacity (WHC) for microbial activity. The pots were placed in the greenhouse at a temperature of 25 ± 2 °C in a completely randomized block design with five replications. Pots were irrigated gently and regularly at 3-day intervals with appropriate amounts of water. Subsequently, the soil's bioactivity was assessed by planting 10 maize seeds directly into the soil every 5 days until reaching 50 days. The plants were harvested 2 weeks after sowing^46,47^. The germination count, shoot length, root length, and total fresh biomass were recorded, and 5 g were collected from each pot for pH and electrical conductivity (EC) measurements. In general, soil chemical analysis was conducted both before and after the decay of plant materials.

### Extraction of phenolic allelochemicals liberated from decayed residues in soil

After 20 days of incorporating invasive weed residues in the soil, the phenolic acids were extracted, whereas, two hundred grams of soil were shaken with 5 mL of distilled water or 0.25 mol/L sodium citrate (pH = 7.0) for 2.5 h^48^. The resulting extracts were centrifuged at 3500 rpm for 20 min, filtered through Whatman 4 filter paper, freeze-dried, and dissolved in methanol (HPLC grade) for determination.

### Quantitative characterization of allelochemicals by LC-DAD/MS analysis

The analysis of phenolic acids involved dissolving ethyl acetate extract and soil phenolic extracts in methanol (HPLC grade) before injection into LC-DAD electrospray ionization (ESI)-MS analysis (Waters, USA) at Ain Shams University, equipped with a DAD detector (Waters Corporation, Milford MA01757, USA). Compounds were separated using a 150 × 4.6 mm C_18_ column. UV/Vis spectra were recorded in the 190–600 nm range and the chromatograms were acquired at 220, 240, 280, 330 and 350 nm. The samples were analyzed by gradient elution at a flow rate of 0.2 ml/min. The mobile phase was a multistep linear solvent gradient system, starting from 100% H_2_O (adjusted to pH 3.2 by HCOOH) up to 100% CH_3_CN in 30 min. The profile and content of phenolic compounds of Hydroxybenzoic acid, cinnamic acid, ferulic acid, coumaric acids, chlorogenic acid, caffeic acid, sinapic acid, vanillic acid, protocatechuic acid, syringic acid, catechin, kaempferol, and quercetin were determined according to the method described previously^49,50^.

### Volatilization of allelochemicals from invasive *E. bonariensis *and *B. pilosa* parts

The shoots of both *E. bonariensis* and *B. pilosa* plants were harvested, and the dried canopy samples were extracted by hydrodistillation^51^. The tested *Z. mays* crop*, C. arvensis*, *P*. *oleracea, *and *E.* crus*-*galli weeds were sterilized and treated with concentrations of 0, 5.0, 10.0, 20 0 µl/ml. Petri dishes were sealed with parafilm and kept at 25 ± 2 °C and then after 7 days seed germination and seedling growth (radical and hypocotyl) were measured. Essential oil was subjected to GC–Ms analysis at The National Research Center. Qualitative identification of the oil constituents was carried out by comparing the retention times and mass fragmentation with computer matching of authentic samples and with published data^52^.

### Statistics analysis

The allelopathic effects of *B. pilosa* and *E. bonariensis* leachates, decaying and volatile compounds on the target plants were compared using the ANOVA test to separate the effect of plant species, concentration and other variations. Where F test indicated significant differences (*P* > 0.05) and followed by Duncan multiple range using SPSS, 19 software (SPSS, Chicago, IL USA). The experimental design was a Complete Randomized Design with four replications and repeated more than one time. Additional data, including EC and pH, were entered for statistical analysis using ANOVA (SPSS, Chicago, IL, USA). Correlation analyses were conducted to test the association between EC, pH, and target plant parameters, serving as response determiners for allelopathic potentials.

### Guidelines of material collections and studies

All the steps of experimentation on three invasive alien species Asteraceae including *Conyza bonariensis* and *Bidens pilosa*, wild weeds, including the collection of plant material, are in compliance with relevant Institutional, National, and International guidelines. The studies were conducted in accordance with local legislation and with permissions from our institutes and complied with the IUCN Policy Statement.

## Results

### *E. bonariensis* and* B. pilosa* spreading and associated weeds relative density in invaded localities

According to surveys, *E. bonariensis* was associated with 16 species of 11 families and achieved an invasion intensity index of 0.221, while *B. pilosa* was associated with 19 species of 11 families and recorded an invasion intensity index of 0.094, resulting in a similarity coefficient of 81.39% within the invaded community. For *E. bonariensis*, *Echinochloa colonum* had the highest relative density, accounting for 11.16% across croplands, orchards, and wastelands in 15 governorates. In the invaded *B. pilosa* community, *Bromus catharticus* had the highest relative density, representing 11.34% in croplands across 5 governorates. The richness R1 and R2 parameters was higher in *B. pilosa* invaded sites compared with invaded sites of *E. bonariensis*. The Simpson index 1 (λ) showed lower diversity in *E. bonariensis* (0.0111) compared to *B. pilosa* (0.034) in invaded sites. However, the Shannon diversity index (H), Pielou’s index (E1), Sheldon index (E2), and Heip’s index (E3) had similar values in invaded sites of both *E. bonariensis* and *B. pilosa*. Finally, the evenness of Hill’s index (E4) and Modified Hill's ratio (E5) was higher in *B. pilosa* (15.46, 30.71) than in *E. bonariensis* (4.62, 8.44) in invaded sites (Table 1).

**Table 1:** Richness, similarity, diversity, and evenness of *E. bonariensis* and *B. pilosa* in invaded localities.

	*Erigeron bonariensis*	*Bidens pilosa*	
Richness			
Richness index 1 (R1)	5.771	6.25	
Richness index 2 (R2)	3.566	3.96	
Coefficient of Similarity *(%)	57.28		
Diversity			
Simpson index1 (λ)	0.0111	0.034	
Shannon index (H')	0.667	0.667	
Evenness (J')			
Pielou’s index (E1)	0.20	0.20	
Sheldon’s index (E2)	0.07	0.07	
Heip’s index (E3)	0.04	0.04	
Hill’s index (E4)	4.62	15.46	
Modified Hill’s ratio (E5)	8.44	30.71	

The invasion level	Invasion intensity index	0.221	0.094
Associate species (Relative Total density %)		1-*Cynodon dactylon* (8.78%), 2-*Phalaris minor* (10.91%), 3-*Euphorbia peplus* (9.71%), 4-*Echinochloa colonum* (5.46%), 5-*Digitaria sanguinalis* (7.58%), 6-*Bromus catharticus* (7.26%), 7-*Cyperus rotundus* (6.38%), 8-*Convolvulus arvensis* (5.46%), 9-*Sonchus* *oleraceus* (5.46%), 10-*Portulaca oleracea* (8.51%), 11-*Chenopodium murale* (7.58%), 12-*Cichorium endivia* (3.93%), *13-Echinochloa crus-galli* (6.66%), 14-*Setaria viridis* (2.00%), 15-*Nidorella aegyptiaca* (2.66%), 16- *Kochia indica* (1.66)	1-*Cynodon dactylon* (6.06%), 2-*Phalaris minor* (6.06%), 3-*Euphorbia peplus* (8.12%), 4-*Echinochloa colonum* (6.96%), 5-*Digitaria sanguinalis* (3.01%), 6-*Bromus catharticus* (11.34%), 7-*Cyperus rotundus* (2.83%), 9-*Convolvulus arvensis* (4.44%), 10-*Sonchus* *oleraceus* (7.67%)**,** 11**-***Portulaca oleracea* (3.73%), 12-*Chenopodium murale* (5.80%), 13-*Cichorium endivia* (1.93), 14-*Malva parviflora* (1.93%), 15- *Medicago polymorpha* (6.95%), 16-*Amaranthus blitum* (4.44%), 17-*Brassica nigra* (4.44%), 18-*Capsella bursa-pastoris* (3.01%), 19-*Sisymbrium irio* (4.44%)
Invaded Localities		Matrouh, Alexandria, Al Behira, Cairo, Giza, Menofia, Qalyubiya, Ismailia, Beni Suef, Minia, Ismailia, Asyut, New Valley, North Sinai, South Sinai	Qalyubiya, Al-Sharkya, Al-Dacahlya, Cairo, and Giza

### Allelopathic potentials of *E. bonariensis* and* B. pilosa* leachates via water extracts

The allelopathic potentials using the extracts of the aboveground parts of *E. bonariensis*, which is widely distributed, were compared with those of *B. pilosa*, which have limited spreading in Egypt using *Z. mays* and their relevant weeds. As for *E. bonariensis* extracts, the most susceptible plant was *P. oleracea*, with EC_50_ values of 2.23, 1.66, and 1.40 (g 100 ml^−1^) for germination, shoot length, and root length, respectively. *C. arvensis* was less susceptible, recording EC_50_ values of 4.71, 4.61, and 3.03 (g 100 ml^−1^) for germination, shoot length, and root length, respectively, to *E. bonariensis* extracts. For *B. pilosa* extracts, *P. oleracea* was the most sensitive, recording EC_50_ values of 2.52, 2.23, and 1.66 (g 100 ml^−1^) for germination, shoot length, and root length, respectively. However, *Z. mays* crop recorded EC_50_ values of (5.52, 4.23, 3.11 g 100 ml^−1^) for *E. bonariensis* and (5.61, 4.4, 3.23 g.100 ml^−1^) for *B. pilosa* in germination, shoot length, and root length, respectively. A significant interaction effect (F = 8.84, *P* ≤ 0.00) of plant species × concentration in *P. oleracea* root length was recorded (Table 2).

**Table 2:** Allelopathic abilities of *E. bonariensis* and *B.pilosa* water extracts based on EC_50_ (g dry wt.100 ml^−1^ water) on different species.

	*Z. mays*	*C. arvensis*	*P. oleracea*	*E. crus-galli*
*E. bonariensis*				
Germination	5.52 ± 0.54	4.71 ± 0.29	2.23 ± 0.12	3.70 ± 1.88
Shoot length	4.23 ± 0.50	4.61 ± 0.67	1.66 ± 0.37	2.69 ± 0.21
Root length	3.11 ± 0.59	3.03 ± 0.26	1.40 ± 0.11	2.12 ± 0.26
*B. pilosa*				
Germination	5.61 ± 0.43	4.83 ± 0.35	2.52 ± 0.99	4.93 ± 0.46
Shoot length	4.40 ± 0.29	4.96 ± 0.34	2.23 ± 0.15	4.02 ± 0.61
Root length	3.23 ± 0.47	3.38 ± 0.43	1.66 ± 0.14	2.37 ± 0.14

	F (*p* value)	F (*p* value)	F (*p* value)	F (*p* value)
Plant type				
Germination	7.89 (0.012)	17.89 (0.00)	127.61 (0.00)	4.90 (0.01)
Shoot length	165.26 (0.00)	7.96 (0.00)	6.30 (0.02)	0.84 (0.33)
Root length	10.71 (0.00)	8.11 (0.00)	8.54.0 (0.00)	26.50 (0.00)
Concentration				
Germination	178.91 (0.00)	167.31 (0.00)	1875.2 (0.00)	101.45 (0.00)
Shoot length	708.70 (0.00)	50.37 (0.00)	75.64 (0.00)	109.65 (0.00)
Root length	62.44 (0.00)	88.94 (0.00)	24.17 (0.00)	387.50 (0.00)
Plant type × concentration				
Germination	2.07 (0.13)	0.788 (0.559)	2.209 (0.10)	0.895 (0.48)
Shoot length	0.66 (0.15)	0.697 (0.603)	2.626 (0.05)	2.45 (0.079
Root length	0.528 (0.71)	2.059 (0.125)	8.84 (0.00)	2.328 (0.091)

### Ethyl acetate crude extract of *E. bonariensis* and* B. pilosa* allelochemicals

Ethyl acetate was used to extract allelochemicals from aqueous solutions. Then, ethyl acetate extract was assessed on *Z. mays*, *C. arvensis*, *P. oleracea*, and *E. crus-galli* seeds and seedling traits that were compared by EC_50_ values. As for, *E. bonariensis* ethyl acetate extract, *P. oleracea* was the most sensitive plant by 89.94, 17.88, 13.02, 58.35 (µg ml^−1^), while, Z. *mays* appeared more tolerable than the other tested plant which recorded EC_50_ by 167.76, 52.27, 40.32 and 164.23 (µg ml^−1^) in germination, shoot length, root length and total biomass fresh weight respectively. As for *B. pilosa* ethyl acetate extracts, *P. oleracea* was the most sensitive plant which recorded EC_50_ values of 77.35, 56.62, 28.81 and 96.9 (µg ml^−1^) in germination, shoot length, root length and fresh total weights respectively, while, *Z. mays* recorded the highest EC_50_ values of 183.23, 66.66, 47.73 and 177.50 (µg ml^−1^) in germination, shoot length, root length and fresh total weights respectively (Table 3).

**Table 3:** Allelopathic abilities of *B. pilosa* and *E. bonariensis* ethyl acetate extracts based on EC_50_ (µg ml^−1^) on different species.

Extracts		*Z. mays*	*C. arvensis*	*P. oleracea*	*E. crus-galli*
*E. bonariensis*	Germination	167.76 ± 1.70	124.59 ± 0.40	89.94 ± 0.60	133.76 ± 1.03
	Shoot length	52.27 ± 0.70	34.56 ± 0.80	17.88 ± 1.50	34.10 ± 0.80
	Root length	40.32 ± 0.23	29.81 ± 0.16	13.02 ± 0.40	30.43 ± 0.13
	Fresh weight	164.23 ± 0.60	114.14 ± 0.70	58.35 ± 0.80	93.09 ± 0.50
*B. pilosa*	Germination	183.23 ± 0.80	103.14 ± 0.47	77.35 ± 0.50	112.09 ± 0.50
	Shoot length	66.66 ± 0.50	66.27 ± 0.60	56.62 ± 0.70	56.23 ± 0.80
	Root length	47.73 ± 0.80	41.10 ± 0.20	28.81 ± 0.40	48.87 ± 0.50
	Fresh weights	177.50 ± 2.71	124.68 ± 1.70	96.90 ± 0.05	111.42 ± 1.60

Sources	*Df*	F (*p* value)	F (*p* value)	F (*p* value)	F (*p* value)
Plant type	1.00	0.10 (0.75)	6.98 (0.01)	11.57 (0.00)	2.45 (0.12)
Traits	3.00	1383.90 (0.00)	1314.76 (0.00)	2067.15 (0.00)	1851.66 (0.00)
Conc	2.00	292.70 (0.00)	495.59 (0.00)	478.74 (0.00)	353.38 (0.00)
Plant type * traits	3.00	0.38 (0.77)	0.99 (0.40)	3.05 (0.03)	0.98 (0.41)
Plant type * Conc	2.00	0.77 (0.47)	1.57 (0.22)	6.95 (0.00)	0.77 (0.47)
Traits * Conc	6.00	38.38 (0.00)	30.59 (0.00)	53.95 (0.00)	43.96 (0.00)
Pant type * traits * Conc	6.00	0.47 (0.83)	0.22 (0.97)	1.45 (0.21)	0.93 (0.48)

*p* values ≤ 0.05.

### Allelopathic potentials of *E. bonariensis *and* B. pilosa* decayed residues in *Z. mays* and* P. oleracea*

**Figure 1:**
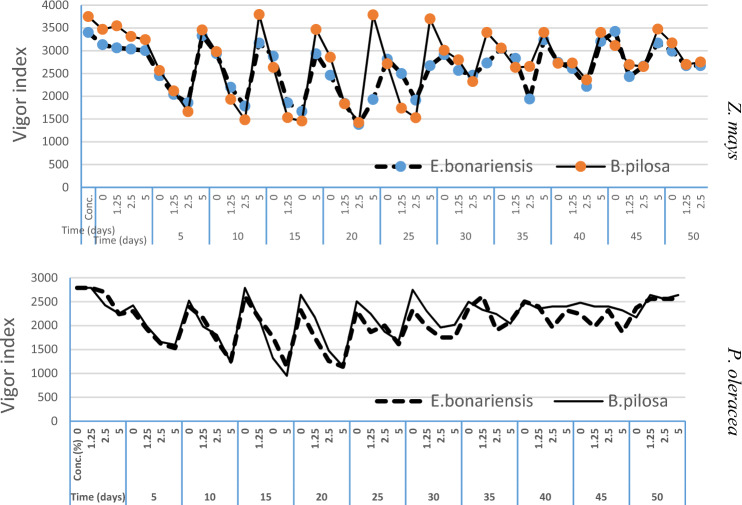
Effect of *E. bonariensis* and *B. pilosa* decayed residues on *Z. mays* and *P. oleracea* vigor index.

The aboveground parts of *E. bonariensis* and *B. pilosa* were decayed in sandy soil for 50 days under the greenhouse to measure their allelopathic potentials against other plants and soil properties. The phytotoxicity analysis differentiated *E. bonariensis* and *B. pilosa* decayed residues, which showed an increase from 5 to 25 days based on the plant species and concentration in vigor index (germination × (shoot length + root length) of *Z. mays* and *P. oleracea* (Fig. 1). As for *Z. mays* response to decayed residues*,* gradually decreasing was recorded in growth traits with a significant interaction in shoot length (F = 5.05, *P* ≤ 0.00) plant species × concentration and (F = 4.37, *P* ≤ 0.00) time × concentration. These interactions were significant in root length (F = 1327.54, *P* ≤ 0.00) concentration and interaction of (F = 3.541, *P* ≤ 0.00) plant species × concentration and (F = 2.48, *P* ≤ 0.02) time × concentration. Also, it was significant in germination (F = 2.576, *P* ≤ 0.03) time, (F = 244.04, *P* ≤ 0.00) concentration and interaction (F = 38.215, *P* ≤ 0.00) plant species × concentration and (F = 6.529, *P* ≤ 0.00) time × concentration respectively. As for *P. oleracea*, in response to decayed residues*,* A gradual decrease was recorded after 10, 15, and 25 days of decayed residues in shoot length, with significant interaction effects observed for plant species × concentration (F = 11.04, *P* ≤ 0.00) and time × concentration (F = 16.73, *P* ≤ 0.00). In root length, there was a significant interaction effect for plant species × concentration (F = 46.79, *P* ≤ 0.00) and time × concentration (F = 2.659, *P* ≤ 0.01). Additionally, in germination, significant interaction effects were observed for plant species × concentration (F = 4.06, *P* ≤ 0.00) and time × concentration (F = 56.815, *P* ≤ 0.00).

**Figure 2:**
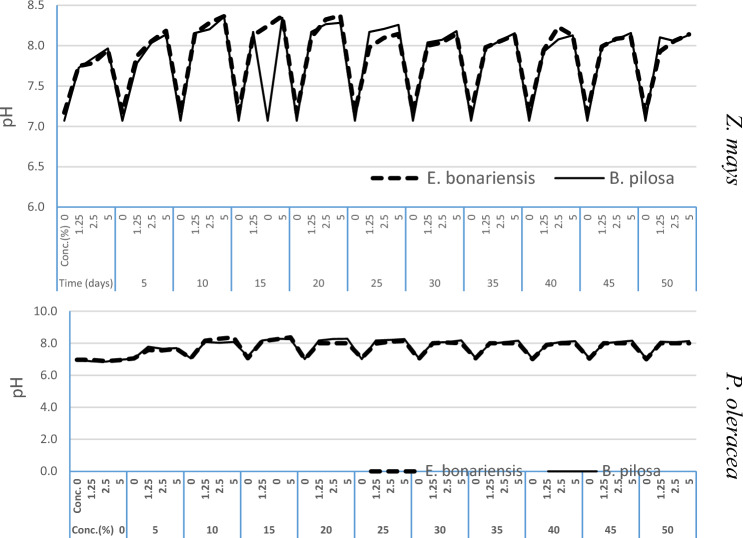
Effect of *E. bonariensis* and *B. pilosa* decayed residues on soil pH values cultivated with *Z. mays* and *P. oleracea*.

**Figure 3:**
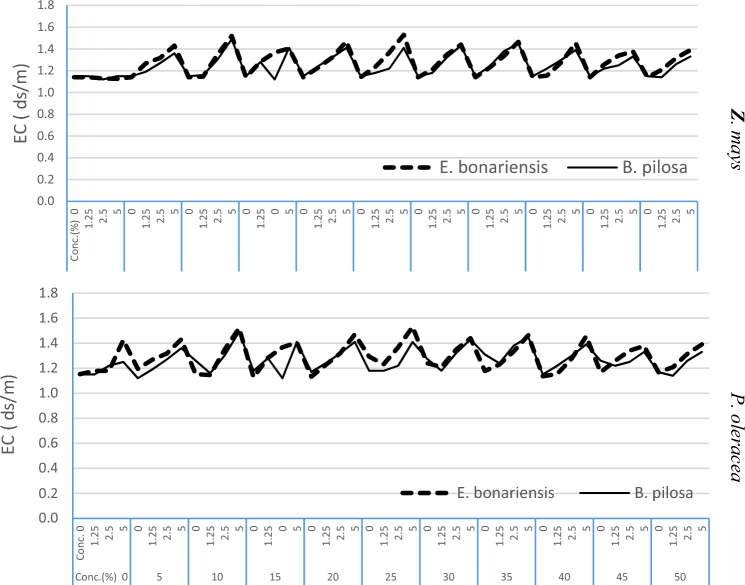
Effect of *E. bonariensis* and *B. pilosa* decayed residues on *Z. mays* and *P. oleracea* soil EC properties.

The decayed materials of *E. bonariensis* and *B. pilosa* showed a significant effect on Z. *mays* soil pH (7.60, *P* ≤ 0.00) time, (10.49, *P* ≤ 0.00), concentration, and interaction (F = 6.82, *P* ≤ 0.00) time × concentration. The decayed materials induced a minor gradient increase from the control to the highest concentration in pH value of *P. oleracea* soil pH (F = 3.80, *P* ≤ 0.00) time, (16.51, *P* ≤ 0.00), concentration, and interaction (F = 3.28, *P* ≤ 0.00) time × concentration respectively (Fig. 2). The decayed materials of *E. bonariensis* and *B. pilosa* in sandy soil cultivated with *Z. mays* and *P. oleracea* had increased soil EC compared to the control, and this increase was proportional to the residue concentrations. There was a significant effect on *Z. mays* soil EC (F = 3.21, *P* ≤ 0.00) in terms of time, (F = 13.25, *P* ≤ 0.00), and concentration, respectively. The decayed materials also showed significant effects on soil EC cultivated with *P. oleracea* concerning from time (F = 4.188, *P* ≤ 0.00) and concentration (12.683, *P* ≤ 0.00), with an increase from the control to the highest concentration (Fig. 3).

### Qualitative and quantitative determination of *E. bonariensis *and* B. pilosa* allelochemicals via leachates and decayed residues

Fourteen phenolic compounds were quantified in the leachates and soil incorporated with decayed materials of the two invasive species using LC/MS (Table 4). Initially, aqueous leachates were extracted by ethyl acetate, dried, and dissolved in methanol for chromatographic analysis. The results revealed that the highest amounts of phenolic acids were chlorogenic acid and caffeic acid, reaching (5.41 and 4.39 µg g^−1^) and (4.53 and 4.46 µg g^−1^) in *E. bonariensis* and *B. pilosa* extracts, respectively. Moderate amounts were observed for hydroxybenzoic, vanillic, gallic, ferulic, protocatechuic, coumaric, sinapic, kaempferol, and cinnamic acids, while protocatechuic acid and catechin were present in low quantities (1.47 and 1.54 μg g^−1^) and (1.27 and 1.91 μg g^−1^) in *E. bonariensis* and *B. pilosa* extracts, respectively.

**Table 4:** Phenolic acids liberated from leachates in water extracted by ethyl acetate (µg g^−1^ crude extract) and decayed residues in soil (µg 200 g^−1^ soil) analyzed by LC-DAD/MS.

Phenolic compounds			Decayed residues (µg 200 g^−1^ soil after 20 days)			Leachates (µg g^−1^)	
No		RT (min)	Control soil	*E. bonariensis*	*B. pilosa*	*E. bonariensis*	*B. pilosa*
1	P-Hydroxybenzoic acid	4.39	0.04	0.84	0.95	3.06	3.43
2	Cinnamic acid	6.14	0.15	0.48	0.42	2.94	2.23
3	Vanillic acid	10.39	0.05	1.73	1.17	3.67	3.55
4	Gallic acids	10.95	0.07	1.17	0.94	3.21	3.17
5	Syringic acid	12.38	0.18	0.62	0.66	2.36	2.11
6	Protocatechuic acid	14.03	0.12	0.32	0.25	1.47	1.27
7	Sinapic acid	15.29	0.22	1.32	0.63	3.76	3.55
8	Chlorogenic acid	16.13	0.00	0.77	0.62	5.41	4.53
9	Caffeic acid	18.02	0.00	0.66	0.53	4.39	4.46
10	Coumaric acid	19.33	0.16	1.66	1.47	3.05	3.62
11	Ferulic acid	20.32	0.13	1.67	1.57	3.85	3.95
12	Catechin	22.0	0.00	0.62	0.43	1.54	1.91
13	Kaempferol	21.23	0.00	0.84	0.61	3.99	4.19
14	Quercetin	23.04	0.00	0.18	0.19	3.41	2.29
	Total		1.12	12.88	10.44	46.11	44.26

Regarding decayed residues in soil samples, the phenolic acids analysis, revealed that coumaric acid and ferulic acid were the predominant compounds with concentrations of (1.66 and 1.67 µg g^−1^) and (1.47 and 1.57 µg g^−1^) in *E. bonariensis* and *B. pilosa* decayed materials, respectively. Additionally, quercetin was detected in relatively low quantities, measuring 0.18 and 0.19% μg g^−1^ in *E. bonariensis* and *B. pilosa* decayed materials, respectively.

### Allelopathic potentials of *E*. *bonariensis *and* B. pilosa *via volatile oils in *Z. mays* and associated weeds

The allelopathic capabilities of *E. bonariensis* and *B. pilosa* volatile oils compounds obtained from hydrodistillation were evaluated on *Z. mays, C*. *arvensis*, *P. oleracea*, and *E. crus-galli*. Based on EC_50_ values, these volatile oils were effective at low concentrations, particularly in the tested species. As for *E. bonariensis* volatile oil, the root length of these plants was the most sensitive trait with EC_50_; 5.86, 3.77, 3.39, and 5.03 (µl ml^−1^) respectively. However, the highest EC_50_ values were displayed from *Z. mays* (the most tolerant plant) by 6.52, 5.86, and 7.85 (µl ml^−1^) in germination, shoot length, and root length respectively. As for *B. pilosa* volatile oils, *P. oleracea* was identified as the most sensitive plant with EC_50_ values of 4.73, 4.82, and 4.10 (µl ml^−1^) in germination, shoot length, and root length respectively. Conversely, *E. crus-galli* displayed remarkable tolerance abilities to *B. pilosa* volatile oils by recording EC_50_ values reaching 7.12, 6.42, and 6.63 (µl ml^−1^) in germination, shoot length, and root length respectively. The interaction effects were significant between oils type and concentration in root length (F = 4.422, *P* ≤ 0.01) *P. oleracea* and (F = 4.819, *P* ≤ 0.014) *C. arvensis* respectively (Table 5).

**Table 5:** Allelopathic abilities of *E*. *bonariensis* and *B. pilosa* volatile essential oils based on EC_50_ (µl ml^−1^) on maize and some associated weeds germination and seedling development.

Essential oils	*Z. mays*	*C. arvensis*	*P. oleracea*	*E. crus-galli*
*E. bonariensis*				
Shoot length	6.520 ± 0.730	4.564 ± 0.600	4.500 ± 1.194	5.220 ± 1.160
Root length	5.868 ± 0.530	3.771 ± 0.410	3.397 ± 1.420	5.037 ± 1.410
Germination	7.853 ± 0.640	4.648 ± 0.430	4.627 ± 1.642	6.590 ± 1.443
*B. pilosa*				
Shoot length	7.324 ± 0.690	5.557 ± 0.390	4.829 ± 1.656	6.422 ± 1.539
Root length	6.224 ± 0.440	4.154 ± 0.390	4. 100 ± 0.895	5.637 ± 1.239
Germination	7.923 ± 0.430	5.273 ± 0.540	4.730 ± 2.111	7.126 ± 1.354

	F (*p* value)	F (*p* value)	F (*p* value)	F (*p* value)
Plant types				
Shoot length	2.721 (0.119)	6.818 (0.019)	0.883 (0.359)	1.036 (0.321)
Root length	7.828 (0.013)	18.375 (0.001)	1.070 (0.313)	4.185 (0.054)
Germination	4.900 (0.042)	5.818 (0.028)	0.000 (1.0)	0.000 (1.0)
Concentrations				
Shoot length	97.879 (0.00)	103.465 (0.00)	416.176 (0.00)	74.499 (0.00)
Root length	242.479 (0.00)	326.042 (0.00)	252.759 (0.00)	82.828 (0.00)
Germination	181.700 (0.00)	117.091 (0.00)	72.895 (0.00)	162.350 (0.00)
Plant type * Conc				
Shoot length	2.052 (0.147)	0.354 (0.787)	0.236 (0.915)	0.317 (0.86)
Root length	1.231 (0.331)	4.819 (0.014)	4.422 (0.01)	1.623 (0.208)
Germination	2.233 (0.124)	1.697 (0.208)	0.263 (0.898)	0.250 (0.906)

### The composition of *E. bonariensis* and* B. pilosa* essential oils by GC/MS

The quantity of essential volatile oils in the dry shoot parts of *E. bonariensis* and *B. pilosa* was quantified to be 0.65% and 0.58% (v/w) respectively. GC/MS analyses identified approximately 37 compounds in the essential oils of these invasive weeds. The major compounds in *E. bonariensis* oils were iso-Caryophyllene (5.2%), β-Farnesene (5.12%), d-limonene (5.12%), and Germacrene (5.08%) respectively. The major constituents of *B. pilosa* oils were 1,8 cineole (5.62%), and α-terpinene (5.43%) followed by trans-sabinene (5.39%) and Camphene (5.11%) respectively as determined by GC/MS (Table 6).

**Table 6:** Composition and percentages of *E. bonariensis* and *B. pilosa* (Shoot parts) essential oils analyzed by GC/MS.

Constituents	*E. bonariensis %*	*B. pilosa%*	Mwt
Camphene	4.71	5.11	136
α-Pinene	1.68	1.90	136
Sabinene	3.48	5.15	136
1-Octen-3-ol	0.63	0.59	128
α-Myrcene	1.64	1.58	136
3-Octanol	0.82	0.76	130
Phellandrene	0.00	4.96	136
α-Terpinene	3.60	5.43	136
O-cymene	2.95	3.59	134
d-limonene	5.12	1.69	136
Trans-sabinene Hydrate	3.29	5.39	155
α-Terpinolene	3.81	4.88	154
L-linalool	2.66	2.83	154
Cis-sabinene Hydrate	1.84	1.22	154
1-Terpineol	2.17	3.17	154
Borneol	1.21	1.12	154
1-4Terpineol	4.60	3.51	154
α-Terpineol	4.22	3.93	154
Thymol	2.95	2.16	150
Iso-thymol	0.70	0.65	150
Carvacryl Acetate	2.84	0.74	150
Trans-caryophyllene	4.15	4.69	204
4-Isopropylidene	1.17	1.08	204
Caryophllene oxide	2.66	3.32	220
Geraniol	3.03	2.79	154
Geraniol Isovalerate	1.43	0.66	138
α-Citronellol	3.44	3.19	156
Camphor	2.15	3.82	152
1,8-Cineole	1.43	5.62	154
Humulene	1.21	1.12	204
Iso-caryophyllene	5.20	2.90	204
α-Cadinol	1.96	2.58	222
Pogostol	1.00	1.50	236
Terpinen-4-ol	0.90	0.68	154
Linalyl Acetate	2.70	0.80	138
Geranyl Actate	2.47	0.51	182
β-Farnesene	5.12	1.31	204
Germacrene	5.08	3.08	204

## Discussion

*E. bonariensis* and *B. pilosa* are two invasive Asteraceae species that differed in the invasion and impacts in cultivated lands of Egypt. Therefore, to understand their detrimental effects we compare their allelopathic effects through leachate by water extraction, decayed residues in soil and volatilization via their essential oils. Also their effects on the invaded ecosystem were evaluated via the ecological index of richness, diversity and evenness. In the studied invaded communities, *E. bonariensis* has lower richness and diversity and evenness as compared with *B. pilosa*. *E. bonariensis* was found in 15 governorates in croplands, orchards, and wastelands, while *B. pilosa* was found in croplands across 5 governorates of Egypt. In contrast, *B. pilosa* was recorded only in cropland habitats^53^. Invasive alien species are a major threat to global biodiversity loss because of their ability to adapt and flourish in diverse environments^54^. Additionally, the allelopathic impacts of these invasive species were evaluated against different species and soil properties, and their allelochemicals were quantified. Invasive weeds may exert a negative impact on other plant species and soil processes driven by allelopathy or nutrient availability^55^ and influence soil physical and chemical properties^56^ and nutrient cycle in the ecosystem^57^. Additionally, Allelopathic substances are proposed as an environment-friendly option to lessen the deterioration of ecosystem services^58^.

### The invasive *E*. *bonariensis *and* B. pilosa* species allelopathic potentials via leachates against varied species

Generally, Based on EC_50_, *E. bonariensis* has greater allelopathic activity than *B. pilosa* through aqueous and organic extracts with varied response patterns in the tested species proportional to the concentrations. According to statistical analysis, there were significant differences in plant species, concentrations and trait response, particularly in seed germination and seedling growth. All plants seem to be leachable (the removal of substances from plants by the aqueous solutes action in different degrees^59^). Phytotoxicity can be attributed to the characteristics of the material^60^. According to ED_50,_ The most sensitive among the four tested plants was *P. oleracea*. However, Z*. mays* was less sensitive to both two extracts. In this respect, the response indices of root length were found to be more susceptible than other measured parameters to the liberated allelochemicals from both extracts. The dose of allelopathic potentials provides valuable biological insights into the invasive impacts of species^61^. Root growth is a more sensitive indicator of phytotoxicity than hypocotyl length^62^. The direct contact between the root and phytotoxic compounds present in the extract might inhibit cell division in the growing root tip^63^. Allelopathy can be an important component of crop/weed interference^64^. Crop plants were more strongly affected by invasive species extracts than weeds^35^.

### The invasive *E. bonariensis *and* B. pilosa* species allelopathic impacts during the decaying process against plant species and soil properties

The invasive species vegetative parts were decayed in the soil to test the biological activity and assess their impacts on the soil parameters. The decayed process displayed a periodic increase over time starting from 5 days and reaching its peaks at 20 and 25 days. Subsequently, a decline in phytotoxic effects was observed from 30 days onwards, diminishing by the end of the decay period (50 days). There were dramatic significant patterns in plant species, concentrations and times. The response pattern of *P. oleracea* was higher than *Z. mays* to decayed allelochemicals. There were slight differences between E*. bonariensis* and *B. pilosa* allelopathic abilities on the tested plant and the soil parameters. Simultaneously, a positive correlation was identified between soil physicochemical properties and the response of plant growth parameters. As for *Z. mays* trails, the vigor index showed a correlation of 0.595 with decayed residue concentration for *E. bonariensis* and 0.538 for *B. pilosa*. Additionally, *Z*. *mays* soil pH exhibited a correlation of 0.758 with decayed residue concentration for *E. bonariensis* and 0.791 for *B. pilosa*. The correlation between *Z. mays* soil EC values and decayed residue concentration was 0.759 for *E. bonariensis* and 0.819 for *B. pilosa*, respectively. As for *P. oleracea* trails, there are positive correlations with the plant vigor index and decayed residue between concentrations (0.433) *E. bonariensis* and (0.055) *B. pilosa* respectively. Similarly, decayed residues exhibited correlations between soil pH and concentration of 0.718 (*E. bonariensis*) and 0.626 (*B. pilosa*) respectively. Additionally, the correlation between soil EC values and decayed residue concentration was 0.595 (*E. bonariensis*) and 0.339 (*B. pilosa)*, respectively. Soil plays a crucial role as a biological environment with the potential to detoxify or toxify allelochemicals through microbial action^65^. The decaying weed residues effects depend upon the release of allelochemicals from them into the soil causing adverse effects on other plants^66,67^. The deleterious effect of decaying weed residues on the growth and yield of subsequent crops in the field was reported^68^. High nutrient availability often observed in plant invasions may be driven in part by the rapid decomposition of exotic plant litter^69^. It is important to identify the allelopathic compounds in soil or water substrates^70^. The most effective allelochemicals have very limited water solubility^71^. Soil incorporation with crop residues resulted in an overall decline in the density and vigor of the weed community^72^. Residue-mediated inhibition can occur only if the susceptibility period of the receptor plant coincides with the inhibitory allelopathic potential peak period^73^. Timing of phytotoxicity is variable, with some reporting it is greatest at early^74,75^ or increasing toxicity with increasing time after incorporation^76^. The changes over time in both the composition and quantity of allelochemicals can either increase or decrease the phytotoxicity^77^.

### *E*. *bonariensis *and* B. pilosa* species allelopathic impacts via volatilization against different species

Here, we test the allelopathic ability of volatile compounds liberated from the studied invasive weeds. The highest amounts of inhibition based on EC_50_ revealed that *E. bonariensis* essential oils had supreme inhibitory effects over *B. pilosa* against the tested plants*.* While, the incidence of growth inhibition was distinguished in the plant species and used concentration as well as the plant traits, and root length was more sensitive than other parameters. Therefore, the allelopathic potential of essential oils demonstrates high inhibition properties towards the selected weeds, compared with the response of the tested crop. These results highlight the influence of invasive plant species volatile oils in the invaded areas. However, these results presented an added value of invasive species essential oil that exhibited weed suppression and can be used as an alternative means to synthetic herbicides. Chemically, invasive plants can modify their environment by releasing secondary metabolites, such as root exudates (liquid) or (gaseous) volatile organic compounds^78^. Allelopathy can regulate plant biodiversity through its impact on plant adaptation, survival, and community organization^79^. However, the effect of allelopathy is not solely harmful; beneficial aspects, such as weed control, are also possible^80^.

### The ways of allelochemicals in the environment and the effects they have on soil and plant communities

Both *E. bonariensis* and *B. pilosa* invasive species demonstrated potent allelopathic efficacy via leachates, decaying, and essential oils on different species, while these allelopathic potentials of aqueous leachates and decayed residues related to phenolic compounds. The Asteraceae family is considered a repository of species to be explored for allelopathy with several associated secondary metabolites such as terpenes, saponins, alkaloids, alkamides, cinnamic acid derivatives, and flavonoids^81^. Phenolic acids are a diverse class of compounds that can act as agents in plant defense^82^. Exotic plants can successfully establish communities due to their relatively strong allelopathic effects in the invaded habitats^83^. Allelopathy of knotweeds may contribute to establishing their new habitats in the introduced ranges as invasive plant species^84^.

The suppressive potential of leachates, decayed residues, and volatilization is influenced by the species, concentration, and response traits of the target species. Stronger detrimental impacts were seen from essential oil followed by leachates compared to decayed residues. Nevertheless, decayed residues displayed a significant negative impact on soil properties, specifically on EC and pH values. However, there are positive correlations between the response of plant parameters and the decayed soil physicochemical properties of EC and pH values. Allelopathy and allelochemicals have provided fascinating insights into plant–plant interactions and their consequences for biodiversity, productivity and sustainability^85^, and could be utilized in conventional or organic agriculture^86^. On the other hand, the high invasion level of *E. bonariensis* joint with allelopathic effectiveness and by low richness and Simpson index 1 (λ) and vice versa in *B. pilosa* species. This allelopathic potentiality proved the strong invasive nature of *E. bonariensis* and impacts on the native plant biodiversity compared to *B. pilosa* species.

## Conclusion

The characteristics of *E. bonariensis* and *B. pilosa* invasive species are revealed by leachates, decomposing residues, and volatile compounds, which are employed as distinct threats to the native species and agricultural soil. The key allelochemicals known to be involved are phenolic compounds via leachates and decayed residues Furthermore, volatile substances were more suppressive than leachates followed by decayed residues. Conversely, *E. bonariensis*, showed highly invasive species and more allelopathic activity than *B. pilosa* species, affecting a wider range of plant species and soil characteristics. Therefore, understanding these allelopathic potentials is crucial for preventing the invasion and impacts on ecosystems and crop productivity and implementing strategic management of invasive species.

## Data Availability

The datasets used during the current study are available from the corresponding author upon reasonable request.
